# A multi-state model analysis of the time from ethical approval to publication of clinical research studies

**DOI:** 10.1371/journal.pone.0230797

**Published:** 2020-03-27

**Authors:** Anette Blümle, Tobias Haag, James Balmford, Gerta Rücker, Martin Schumacher, Nadine Binder

**Affiliations:** 1 Institute for Evidence in Medicine (for Cochrane Germany Foundation), Faculty of Medicine and Medical Center, University of Freiburg, Freiburg, Germany; 2 Institute of Medical Biometry and Statistics, Faculty of Medicine and Medical Center, University of Freiburg, Freiburg, Germany; 3 Institute for Prevention and Cancer Epidemiology, Faculty of Medicine and Medical Center, University of Freiburg, Freiburg, Germany; 4 Institute of Digitalization in Medicine, Faculty of Medicine and Medical Center, University of Freiburg, Freiburg, Germany; Institute for Quality and Efficienty in Health Care (IQWiG), GERMANY

## Abstract

**Background:**

Results of medical research should be made publicly available in a timely manner to enable patients and health professionals to make informed decisions about health issues. We aimed to apply a multi-state model to analyze the overall time needed to publish study results, and to examine predictors of the timing of transitions within the research process from study initiation through completion/discontinuation to eventual publication.

**Methods:**

Using a newly developed multi-state model approach, we analysed the effect of different study-related factors on each of the transitions from study approval to eventual publication, using a data set of clinical studies approved by a German research ethics committee between 2000 and 2002.

**Results:**

Of 917 approved studies, 806 were included in our analyses. About half of the clinical studies which began were subsequently published as full articles, and the median time from study approval to publication was 10 years. Differences across model states were apparent; several factors were predictive of the transition from study approval to completion, while funding source and collaboration were predictive of the transition from completion to publication.

**Conclusions:**

The proposed multi-state model approach permits a more comprehensive analysis of time to publication than a simple examination of the transition from approval to publication, and thus the findings represent an advance on previous studies of this aspect of the research process.

## Introduction

Results of medical research should be made publicly available in a timely manner to enable patients and health professionals to make informed decisions about health issues [1]. However, there are several barriers inhibiting the translation of research knowledge into practice. Firstly, a large proportion of clinical studies are prematurely discontinued [2–9]. Secondly, not all research findings are ultimately published in peer-reviewed journals [10]. A systematic review of the extent of non-publication of studies approved by research ethics committees (REC) or included in trial registries showed that only about half of all studies were eventually published in a peer-reviewed journal [8, 11, 12].

A Health Technology Assessment report found that the main reason given by investigators for not publishing their studies was ‘lack of time or low priority’, followed by ‘results not important enough’ and ‘journal rejection’ [13, 14]. Lack of financial and personnel resources are also frequently-cited reasons for non-publication [15]. Specific study-related factors are also associated with whether or not a study is published, particularly the direction of the study results [11]. “Negative” study results, e.g. no significant difference between treatment arms, are less likely to be published than “positive” results, i.e. significant effects favouring the experimental treatment [16–20]. This is also associated with time to publication, with positive results published on average about 1–3 years more quickly than negative results [17–19, 21, 22]. Source of funding is also important; negative results are more likely to be published when studies are funded by non-profit institutions than when they are commercially funded [23]. In a cohort of biomedical research studies approved by a German REC we showed that multicentre and international collaboration, large sample size, a declared study funding source and the sponsor’s involvement in the study were also positively associated with subsequent publication [24, 25].

The ‘selective publication’ of studies results in a restricted and frequently over-optimistic body of evidence. Treatments are often erroneously accepted as effective, whereas null or adverse effects are underestimated. The problem is magnified when studies are combined in meta-analyses or systematic reviews, and can lead to inappropriate health care recommendations and ultimately non-optimal treatment that can prolong patients’ suffering, cause harm and in the worst case, cost lives [12, 26, 27].

**Fig 1:**
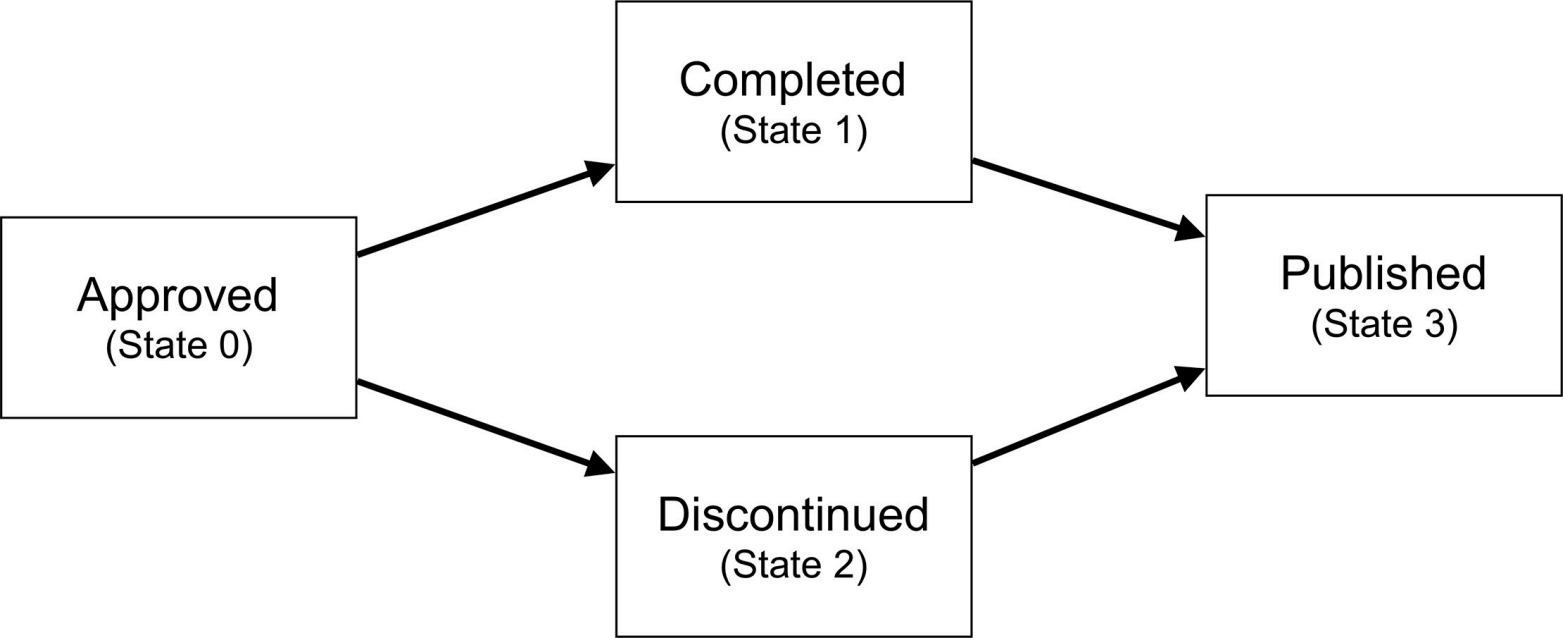
Illustration of the four-state model. The boxes indicate the states, the arrows the possible state transitions.

The process of conducting and publishing a study can be divided into multiple event types or “multi-states” occurring over a period of time. All studies start in an initial state, e.g. REC approved, may pass through one or more intermediate states, and may finally result in an absorbing state, e.g. published. The process is typically visualized by a directed graph, often called a multi-state model, where the states are drawn as nodes and the possible state transitions are indicated by arrows (Fig 1). For studies that have begun, two intermediate states are possible: a) the study is completed, i.e. the study has ended as planned and participants are no longer being followed up; or b) the study is discontinued (e.g. was stopped early due to adverse events). Either of these scenarios can then lead to eventual publication of the findings. This four-state model, originally developed in a Master’s thesis written by one of the co-authors (TH) [28], allows for the systematic examination of variations in the total time from ethical approval to publication, distinguishing between the time required to conduct the study (i.e., study initiation to either study completion or discontinuation), and the time required to analyse, write up and publish the study findings (i.e., study completion/discontinuation to publication).

To demonstrate this novel analytic approach we conducted a secondary analysis of a data set collected for a previous investigation of study protocols submitted to a German REC [24]. In Germany (like in many countries), registration in a clinical trial registry is not mandatory, but all studies, regardless of their design, are required to be approved by an REC before they can legally begin. Using the aforementioned multi-state model, we analysed the effect of different factors on each of the illustrated transition intensities within the research process from study approval to eventual publication.

## Methods

### Study design and data

The data set we used to demonstrate our newly developed four-state model approach included all research protocols of human research studies submitted to and approved by the University of Freiburg REC between 2000 and 2002 [24]. The design of each study was determined according to predefined criteria (S1 Fig). Study characteristics were extracted by a research assistant (FV or AH, see acknowledgments). In cases of uncertainty, the issue was discussed with the lead author to reach a consensus. The lead author cross-checked the other database entries. Subsequent publications were identified via electronic literature searches (AH, PO) and a survey sent to the applicants (AH, AB, actively supported by the REC). The study was piloted by investigating protocols approved by the REC in 2000. The search for publications was conducted in 2006, the survey to applicants was sent in 2007 (followed by a reminder three months later) asking them to confirm the publications that had been identified and to add further publications. It was also asked whether the project (a) had been completed as planned, (b) had been discontinued, (c) had not been started at all or at the local study site, or (d) was still ongoing, and if so its current status (i.e., recruitment closed, data collection completed, or preliminary results published) [24]. The results of the pilot project were published in 2008 [25]. This was then complemented by protocols approved in 2001 and 2002. For those studies, the search for publications took place between August 2009 and January 2010, and surveys were sent in February 2010. For the pilot year an updated search was carried out between July 2011 and January 2012 (S2 Fig).

For the present study, in order to comply with the survey information received, we defined the study initiation date as the *year* of REC approval, the study completion date as the *year* in which data collection was completed, the study discontinuation date as the *year* in which the study was discontinued, and the study publication date as the *year* of electronic publication of the first manuscript presenting findings of the study.

Data from REC files, incl. application, protocol, correspondence with the REC, and from the survey were recorded for several covariates potentially associated with the occurrence of state transitions. These were: (a) whether the study was an RCT (y/n); (b) sample size; (c) funding source (commercial, non-commercial or not reported); (d) industry involvement in the conduct and/or analysis of the study (y/n); (e) a composite variable of number of study centres and international collaboration (categorized as ‘multi-centre, international collaboration’, ‘multi-centre, national collaboration’ and ‘single centre’); and (f) whether the study had a primary outcome (y/n). The only covariate for which there was a non-trivial amount of missing data was sample size; for these 37 (4.6%) studies the median number of participants (n = 120) was imputed.

For two covariates, funding source and industry involvement, information was also extracted from the final publication. There was occasional inconsistency between the REC protocol and the publication, likely because it was not an explicit requirement for the protocol to state the study funding source or (anticipated) involvement of the sponsor. In these cases we took the pragmatic approach of designating studies as (non-)commercially-funded if (non-)commercial funding was mentioned either in the protocol or the publication; the same applies for industry involvement.

### Statistical analysis

We aim to analyze the time needed to publish study results, and to estimate predictors of the timing of transitions within the research process from study initiation through completion/discontinuation to eventual publication. To accomplish this, we employ time-to-event approaches, which make use of the information that (or whether) a certain event occurs and the time until which the transition to this event occurs. Thereby, the multi-state model [29] is central. Statistical inference is made on the basis of the transition intensities (hazards), the instantaneous risk of moving from one state to another. Even though they were developed for situations where transitions are observed in continuous time (potentially subject to right-censoring), multi-state model approaches can be used to deal with more challenging observation patterns, e.g., interval censoring [30], based on the assumption that patterns of missing time-to-event information are *non-informative* for the event history process. In our case, the observation pattern is retrospective event collection in a prospective cohort, which we have outlined along with the corresponding censoring mechanisms in the S1 Appendix and S1 Table.

We first investigated a simplified two-state model with only one possible transition, Approved → Published, corresponding to a standard survival analysis. We fitted the non-parametric Kaplan-Meier estimator to estimate the cumulative distribution function of total time to publication and the Cox model to estimate potential effects of the covariates on the hazard of publication using the R package survival. From the estimation procedure, we further obtained the median time to publication incl. corresponding confidence intervals based on the Brookmeyer-Crowley approach [31, 32].

Our main analysis was based on the four-state model, where we used numerical estimators based on the likelihood function to estimate covariate effects on each transition intensity from interval-censored data. We assumed piece-wise constant baseline intensities, allowing us to compute the full likelihood analytically (see section 2 in S1 Appendix). Maximization of the log-likelihood used the Method of Moving Asymptotes [33]. All covariates in the multivariable analyses were entered simultaneously, and no interactions were hypothesized.

The function to estimate the transition intensities as well as covariate effects was written in C by one of the authors (TH) and can be called from R [34] via a shared library object. Mathematical details as well as documentation of the function can be found in the appendix of [28].

## Results

### Baseline and outcome characteristics

**Fig 2:**
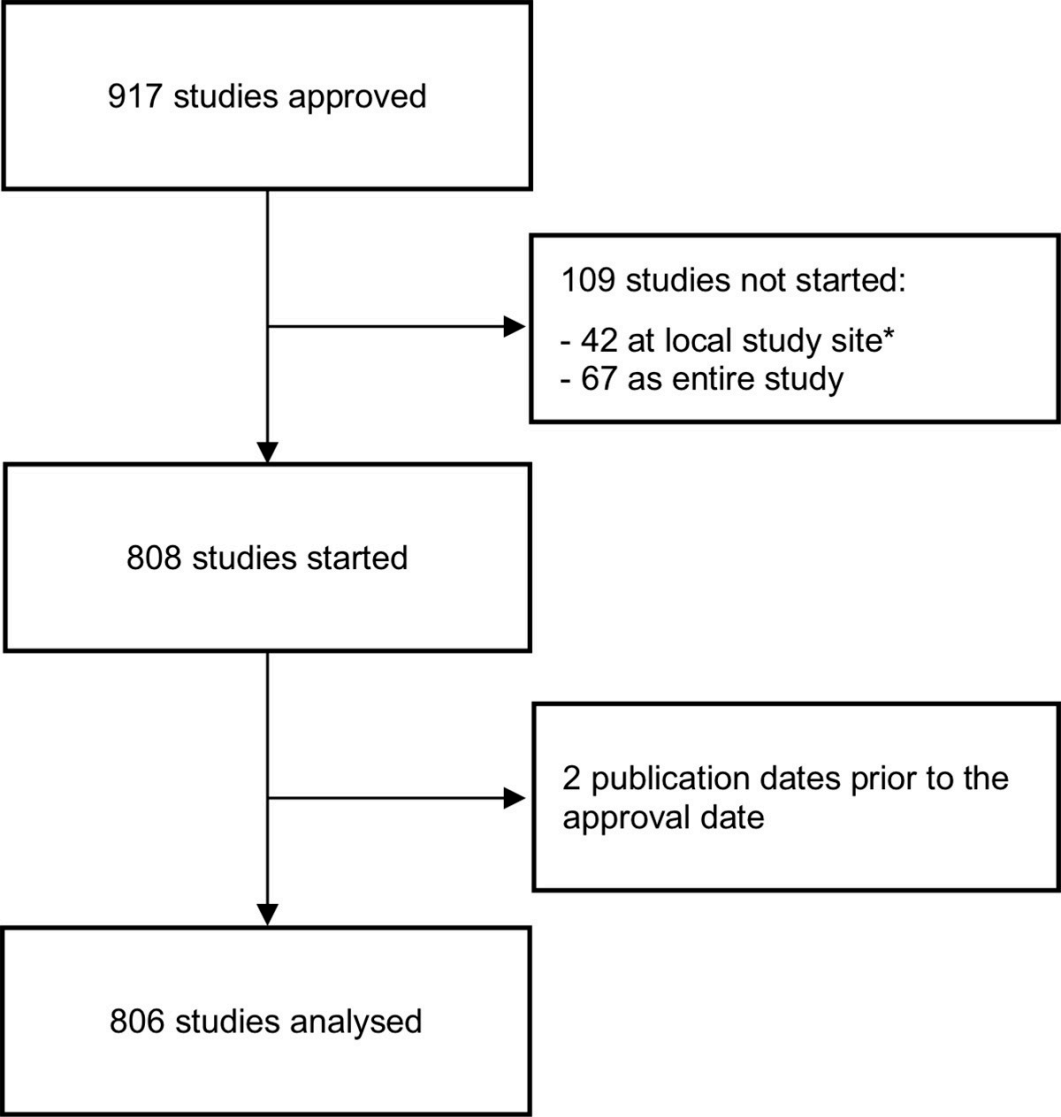
Flowchart of study protocols approved between 2000 and 2002 by the research ethics committee of the University of Freiburg/Germany. *We included one additional study compared to our previous analyses [24], because it later turned out that the survey results were incorrect (study started at local study site).

Of 917 approved studies, 806 were eligible for inclusion in our analyses (Fig 2).

Study characteristics are displayed in Table 1.

**Table 1:** Baseline characteristics of included studies (n = 806).

Characteristic	
**Sample size**	
Mean (sd)	386 (909)
Median	120
Range	3–3900
**Study design (n, %)**	
RCT	354 (43.9)
Other	452 (56.1)
**Collaboration**	
Multi-centre, international	189 (23.5)
Multi-centre, national	275 (34.1)
Single-centre, national	342 (42.4)
**Primary outcome present (n, %)**	
Yes	482 (59.8)
No	324 (40.2)
**Funding (n, %)**	
Commercial	357 (44.3)
Non-commercial	217 (26.9)
Not reported	232 (28.8)
**Industry Involvement (n, %)**	
Yes	348 (43.2)
No	458 (56.8)

**Fig 3:**
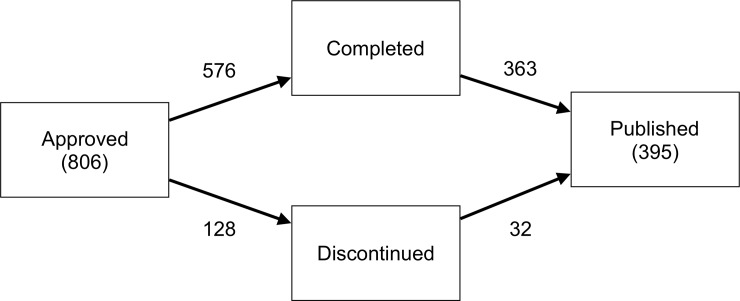
Four-state model. The numbers next to the arrows indicate the total numbers of studies for each transition.

The overall survey response rate was 91%. By 2010 / 2012, 576 (71%) studies were completed and 128 (16%) had been discontinued. A further 41 were classified as ‘ongoing’ based on survey responses, while for 61 studies the status was unclear (i.e., no survey response, and no evidence of publication). Of the completed studies 363 (63%) had been published as a full article, as had 32 (25%) of the discontinued studies. Overall, 49% of studies had been published by 2010 / 2012. Fig 3 displays the number of studies observed to enter each state within the four-state model.

For the intermediate states of study completion and discontinuation, time-to-event information was only available in 23% and 13% of cases respectively, thus the majority of observations were interval-censored [35]. S1 Table summarizes the different observation cases within the four-state model with potential interval- or right-censoring and reports the corresponding counts and percentages in the data set.

### Two-state model

The median time overall was 10 years (i.e., after 10 years half of all studies had been published) with an approximate 95% confidence interval of [8, ∞], and 4 years (95% CI [4, 5]) for studies that were actually published. The latter, however, is an underestimate of the true time to publication as it is biased by ‘conditioning on the future’ [36]; that is, only including studies for which the outcome was successful.

**Fig 4:**
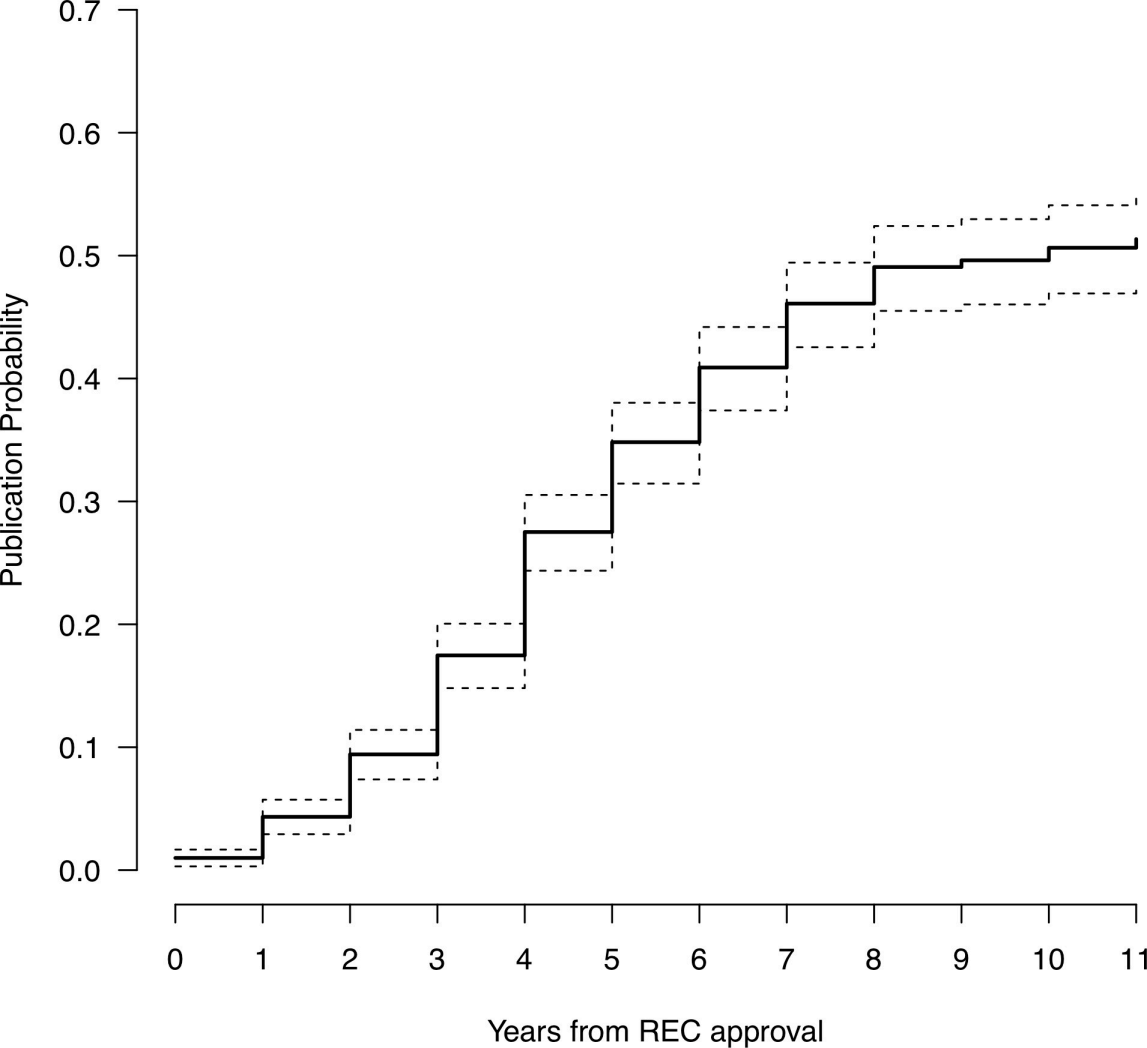
Complement of Kaplan-Meier estimate with its 95% confidence interval of the cumulative distribution function of the total time to publication. Time point 0 is the REC’s positive vote, i.e. study approval.

The publication rate, i.e., the probability that a previously unpublished study is published, is highest four to seven years after REC approval. Studies that have not been published after nine years are very unlikely to be subsequently published (publication probability < 10%) (Fig 4).

Table 2, displaying the findings of the two-state model predicting the transition from approval to publication, shows that non-commercially funded studies were published faster than both commercially-funded studies and those in which the funding source was not reported. Studies with industry involvement in the conduct or analysis were published faster than those without industry involvement, as were studies with a primary outcome. Finally, single-centre studies tend to be published faster than multi-centre studies with an international partner. We note here that the variance inflation factors from the covariance matrix of the parameter estimates in the two-state Cox model were overall below three, i.e., there are probably no strong issues due to multicollinearity.

**Table 2:** Estimated covariate effects with their 95% confidence intervals (two-state model).

	Hazard ratio	95% CI
**Approved → Published**		
log Sample Size	1.038	[0.959, 1.123]
RCT vs. other	0.884	[0.693, 1.128]
Funding: Commercial vs. non-commercial	0.337	[0.246, 0.461]
Funding: Not reported vs. non-commercial	0.298	[0.221, 0.404]
Industry involvement: Yes vs. no	1.829	[1.302, 2.567]
Primary outcome: Yes vs. no	1.365	[1.041, 1.790]
Collaboration: Multi-centre, national vs. single	0.900	[0.648, 1.249]
Collaboration: Multi-centre, international vs. single	0.619	[0.454, 0.844]

### Four-state model

We now consider each transition in the four-state model in detail. As shown in Table 3, several factors predicted study completion. Funding source was strongly predictive of subsequent study completion; non-commercially-funded studies were completed faster than commercially-funded studies and those in which the funding was unstated. Studies in which the sponsor was involved in the conduct or analysis were completed considerably faster than others. Also, studies with a predefined primary outcome tended to be completed faster than others. The main factors predicting discontinuation included study design and sample size: the hazard for an RCT to be discontinued after approval was higher than that for a non-RCT type study, while studies with a smaller sample size were also more likely to be discontinued. Funding source also tended to be predictive (borderline significant), with commercial funding or unstated funding increasing the hazard for study discontinuation as compared to non-commercial funding. Once completed, funding source was strongly predictive of subsequent publication; again, non-commercially-funded studies were published faster than commercially-funded studies and those in which the funding was unstated. Single-centre studies were published faster after completion than multi-centre/international studies. Prediction of publication following discontinuation was limited by the low number of studies in this category (n = 32), which led to wide confidence intervals around some of the estimates. However, among discontinued studies those with a larger sample size tended to be published considerably faster than others, whereas the hazard for an RCT to be published after discontinuation was considerably lower than that for a non-RCT type study.

**Table 3:** Estimated covariate effects with their 95% confidence intervals (four-state model).

	Hazard ratio	95% CI
**Approved → Completed (n = 576)**		
Log sample size	0.972	[0.951, 0.993]
RCT vs. other	1.077	[0.855, 1.358]
Funding: Commercial vs. non-commercial	0.523	[0.359, 0.762]
Funding: Unstated vs. non-commercial	0.648	[0.485, 0.866]
Industry: Involved vs. not involved	2.294	[1.595, 3.298]
Primary outcome: Yes vs. no	1.422	[1.102, 1.836]
Collaboration: National multi-centre vs. national single-centre	0.848	[0.614, 1.171]
Collaboration: International multi-centre vs. national single-centre	0.694	[0.521, 0.924]
**Approved → Discontinued (n = 128)**		
Log sample size	0.857	[0.819, 0.897]
RCT vs. other	2.375	[1.529, 3.689]
Funding: Commercial vs. non-commercial	1.795	[1.001, 3.220]
Funding: Unstated vs. non-commercial	1.565	[1.065, 2.301]
Industry: Involved vs. not involved	1.093	[0.541, 2.208]
Primary outcome: Yes vs. no	1.532	[0.924, 2.542]
Collaboration: National multi-centre vs. national single-centre	0.751	[0.429, 1.316]
Collaboration: International multi-centre vs. national single-centre	0.815	[0.482, 1.379]
**Completed → Published (n = 363)**		
Log sample size	1.086	[0.999, 1.179]
RCT vs. other	0.832	[0.620, 1.117]
Funding: Commercial vs. non-commercial	0.429	[0.286, 0.644]
Funding: Unstated vs. non-commercial	0.374	[0.254, 0.551]
Industry: Involved vs. not involved	1.124	[0.716, 1.766]
Primary outcome: Yes vs. no	0.998	[0.711, 1.400]
Collaboration: National multi-centre vs. national single-centre	0.825	[0.527, 1.290]
Collaboration: International multi-centre vs. national single-centre	0.597	[0.392, 0.908]
**Discontinued → Published (n = 32)**		
Log sample size	1.481	[1.468, 1.495]
RCT vs. other	0.275	[0.105, 0.724]
Funding: Commercial vs. non-commercial	0.504	[0.163, 1.559]
Funding: Unstated vs. non-commercial	--	--
Industry: Involved vs. not involved	0.437	[0.142, 1.338]
Primary outcome: Yes vs. no	1.988	[0.411, 9.625]
Collaboration: National multi-centre vs. national single-centre	2.397	[0.501, 11.481]
Collaboration: International multi-centre vs. national single-centre	1.790	[0.507, 6.317]

### Sensitivity analyses

Given the high proportion of missing information on the timing of model transitions, analyses were repeated using logistic regression. The outcomes were successful completion of each state transition, without taking into account time to event. Overall the results of the logistic regression analysis predicting transition from approval to publication show no discrepancies to the results of the two-state model (S2 Table).

The results (S3 Table) were similar to those obtained in the multi-state model analysis, but with larger standard errors. Funding source emerged as strongly predictive of the transition from approved to completed, with both commercially-funded studies and those in which the funding was unstated less likely to be completed than non-commercial studies. Moreover, two additional covariates emerged as predictive of the transition from completed to published: a pre-specified primary outcome, and industry involvement. With these changes, predictors of the transition from approved to completed were largely identical to those of the transition from completed to published.

## Discussion

In this study, we applied a newly-developed four-state model to examine the research process from ethical approval to publication among clinical studies approved by a German REC between 2000 and 2002. We determined the overall time needed to publish the study results, distinguishing between the time required to conduct the study (i.e., study initiation to either study completion or discontinuation), and the time required to analyse, write up and publish the study findings (i.e., study completion/discontinuation to publication). We also examined predictors of the timing of transitions within the research process as described by the four-state model, i.e. from study initiation through the completion/discontinuation of data collection to eventual publication. About half of initiated clinical studies were subsequently published as full articles by 2010 / 2012, with the median time from study approval to publication 10 years. Discontinued studies were less often (25%) published than completed studies (63%). There is little consensus in the literature regarding time to publication, with wide variation depending on the cohorts investigated [3, 15, 37–39]. Previous evaluations, however, excluded studies with unknown completion date, which likely led to underestimation of time to publication [37].

The primary findings of this study were that a number of study-related factors which predicted transitions in the conduct of research studies from initiation to eventual publication could be identified, and that these factors differed across the transitions specified in the four-state model. The latter finding demonstrates the value of differentiating the time required to collect data from the time required to analyse and write up the findings for publication. The use of a four-state model permits a more comprehensive analysis of the research process than is possible if one simply examines the period from approval to publication in a single analysis. It thus represents an advance on previous studies of time to publication [11].

The hazard from completion to publication was mainly predicted by funding source, with the findings consistent with previous research finding industry-funded studies to have a lower publication rate than studies funded by medical centres [38]. This may suggest that commercial funders have lower incentive to publish findings in academic journals. Interestingly, Goldacre et al recently found that among clinical trials registered in the European Union clinical trials registry, those with a commercial sponsor were more likely to include result data in the registry [40]. For commercial funders, journal publication may not always be the preferred means of communicating study findings to the wider community. It may also point to characteristics of the findings, e.g. whether or not the main hypotheses were supported, influencing the decision whether or not to publish to a greater extent for commercially-funded studies, as has been found previously [11]. The influence of funding source is also reflected in the transition from approved to discontinued, where commercial studies were more likely to be discontinued. Again, this is suggestive of emerging study findings influencing the decision whether or not to continue a study to a greater extent if the study is commercially funded.

Consistent with Ioannidis [17], we did not find sample size affected overall time to publication in the two-state model analysis, and in the four-state model analysis sample size was related only to the time from approval to discontinuation, with smaller studies more likely to be discontinued than larger studies.

The major limitation of our study was that in over three-quarters of studies the year of completion or discontinuation was unavailable. As it was not a focus of investigation at that time, survey respondents were not asked to specify the year in which the study was completed or discontinued. Rather, this information was sourced from annual reports submitted to the REC, where it was inconsistently reported. The underlying data set was, therefore, insufficient to perform an adequate time-to-event analysis (with 2 or more states). However, the method we used to maximise the log-likelihood in the time-to-event analyses, the Method of Moving Asymptotes [33], has been extensively studied with simulated data assuming different underlying baseline intensities and a similarly large amount of missing information on the timing of intermediate states still resulting in almost unbiased covariate effect estimates [28]. It is also important to note that we were mainly missing only time-to-event data for these state transitions, rather than whether or not the study was completed or discontinued. It is reassuring that the sensitivity analyses we performed, which did not use time-to-event information, mostly resulted in similar findings to the primary analyses with regard to the predictors of state transitions. Moreover, while model estimates in the time-to-event analyses had wider confidence intervals than we would have had with full data, even with limited data the estimates were more precise than in the logistic regression analyses, which did not take into account time-to-event data. Despite the presence of missing time-to-event data, Cox regression is preferable to logistic regression because of the long follow-up period we considered and the large heterogeneity in follow-up times between our two cohorts. With extended follow-up, the odds ratio is a poor estimate of the hazard ratio, tending to overestimate the risk factor effect particularly when there are many observed events [41].

A further limitation was that the exact timing of events was often not determinable. One problem is that it is not always easy to define the completion date of a study. For example, the study completion date may be the date on which the last patient completed the final follow-up, the date on which the database was closed, or the date of the study end report, e. g. final report to the funder. Therefore, we decided not to consider specific dates, but rather to restrict our analysis to the year in which events occurred. We note here that instead of fitting a continuous-time model, a model for time-discrete or grouped time-to-event data would also have been an adequate choice towards fitting this kind of data observed in time periods [42]. Still, our approach can be seen as a suitable approximation. Since REC approval always occurs prior to data collection, this would have had the effect of increasing the estimate of the time required from study initiation to completion/discontinuation. In most cases, we expect the difference would be minimal or non-existent, however it is conceivable that studies of complex behavioural interventions may require a considerable period of intervention development work before participants can be recruited. Additionally, while the 2000 cohort of studies was followed over 11 years, the 2001/2002 cohort was only followed until 2010, see S2 Fig. This reduced the number of studies from these cohorts which we were able to identify, which may have led to an overestimate of the median time to publication. However, this would not lead to a systematic bias if the assumption of independency between the publication process and the censoring mechanism is not violated. There were also a number of studies for which the planned sample size was unknown. Imputing the median sample size likely led to underestimating the variance of this predictor, thus reducing our capacity to find an effect. Finally, only a few trials were published following discontinuation, and confidence intervals for the effect estimates for this transition were partly broad (indeed, no estimate could be computed for the comparison between non-commercially funded studies and funding unstated). These findings should therefore be interpreted with caution.

We believe that poor availability of such data is typical, at least from 2000–2002 in Germany. Things have since changed for the better, particularly due to the founding of the German Registry of Clinical Trials (DRKS) https://www.drks.de/drks_web/ in 2008. Study investigators who apply for an ethics approval are now requested to register there, such that a study is identifiable by a unique study ID. This is intended to ensure it is easier to track studies over time, allowing more reliable data on time to publication to be collected.

Finally, we are obliged to call attention to the importance of careful data collection. Many of the limitations of this study we have discussed are a direct consequence of our failure (in retrospect) to ask the right questions in the survey to ensure we could establish the existence and timing of state transitions. Readers who are considering undertaking a multi-state model analysis should ensure that a data analysis plan is drafted before data collection begins, to help prevent important factors in the analysis being overlooked.

## Conclusions

The use of a four-state model permitted a comprehensive examination of the research process, allowing for the identification of predictive relationships between states that were not apparent from a simple two-state analysis. While the presence of missing time-to-event information was an important limitation, it did not appear to have an undue influence on the findings. Since this study was initiated, the proportion of studies which are registered in a clinical trial registry prior to data collection has greatly increased [43], and therefore future investigations of time to publication should have access to more complete information regarding the timing of state transitions. We recommend using modern statistical methods of event history analysis (multi-state models) to permit a detailed study of the publication process in medicine, epidemiology, or other areas of research.

## Supporting information

S1 Fig Criteria for study design classification. (TIFF)

S2 Fig Timeline of study research activities. (TIFF)

S1 Appendix Extended statistical information. (DOCX)

S1 Table Four-state model observation cases with coding of potential censoring times and corresponding counts and percentages in the data set. (DOCX)

S2 Table Estimated covariate effects with their 95% confidence intervals from a logistic regression model (see Sensitivity analysis in main manuscript). (DOCX)

S3 Table Estimated covariate effects with their 95% confidence intervals from separate logistic regression models (see Sensitivity analysis in main manuscript). (DOCX)
