# Supplementary figures and images for: A multi-state model analysis of the time from ethical approval to publication of clinical research studies
Source: PLoS One. 2020 Mar 27;15(3):e0230797. doi: 10.1371/journal.pone.0230797 (PMC7100954; doi:10.1371/journal.pone.0230797)

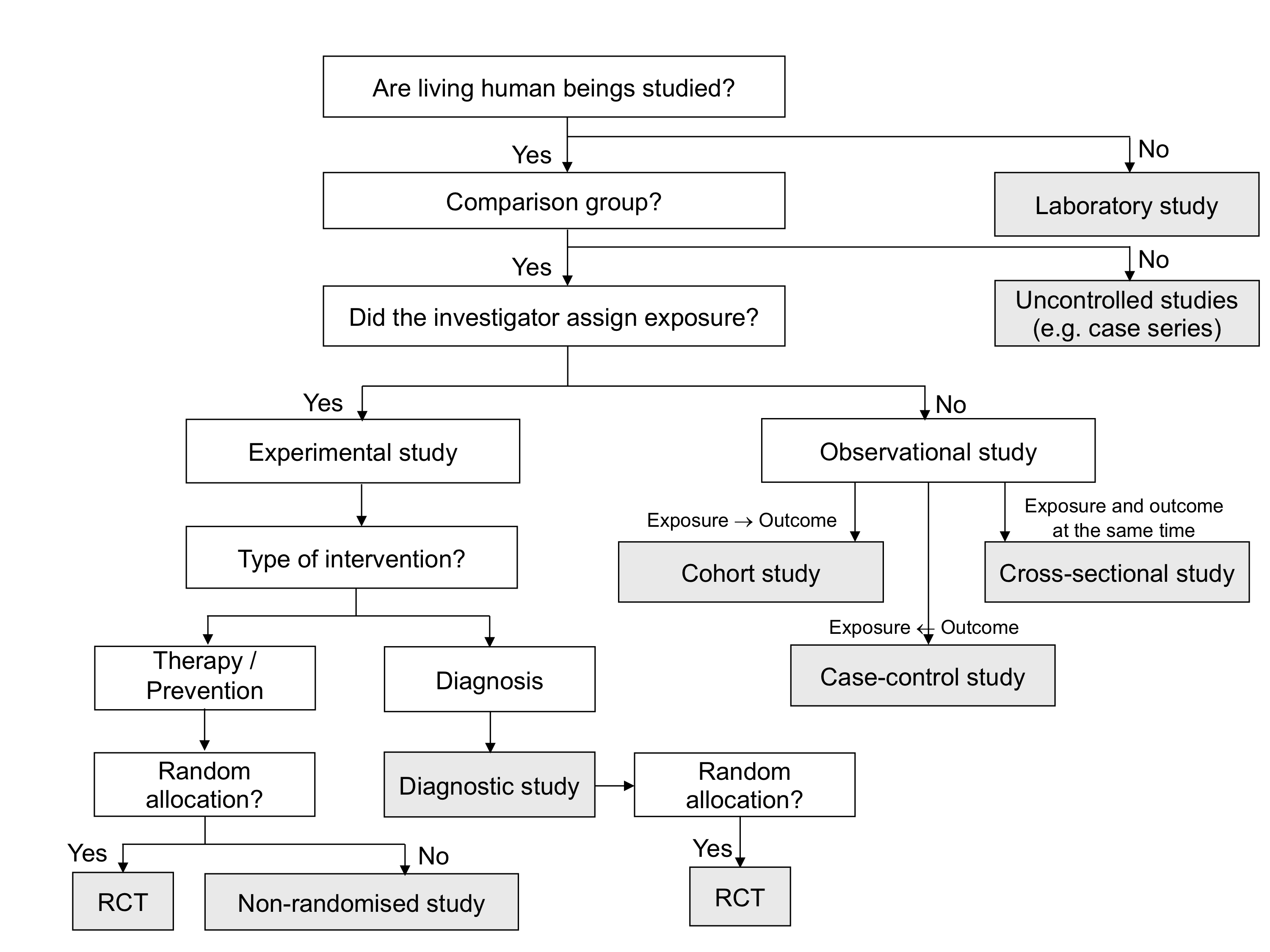

Supplement: S1 Fig — (TIFF) [file pone.0230797.s001.tiff]

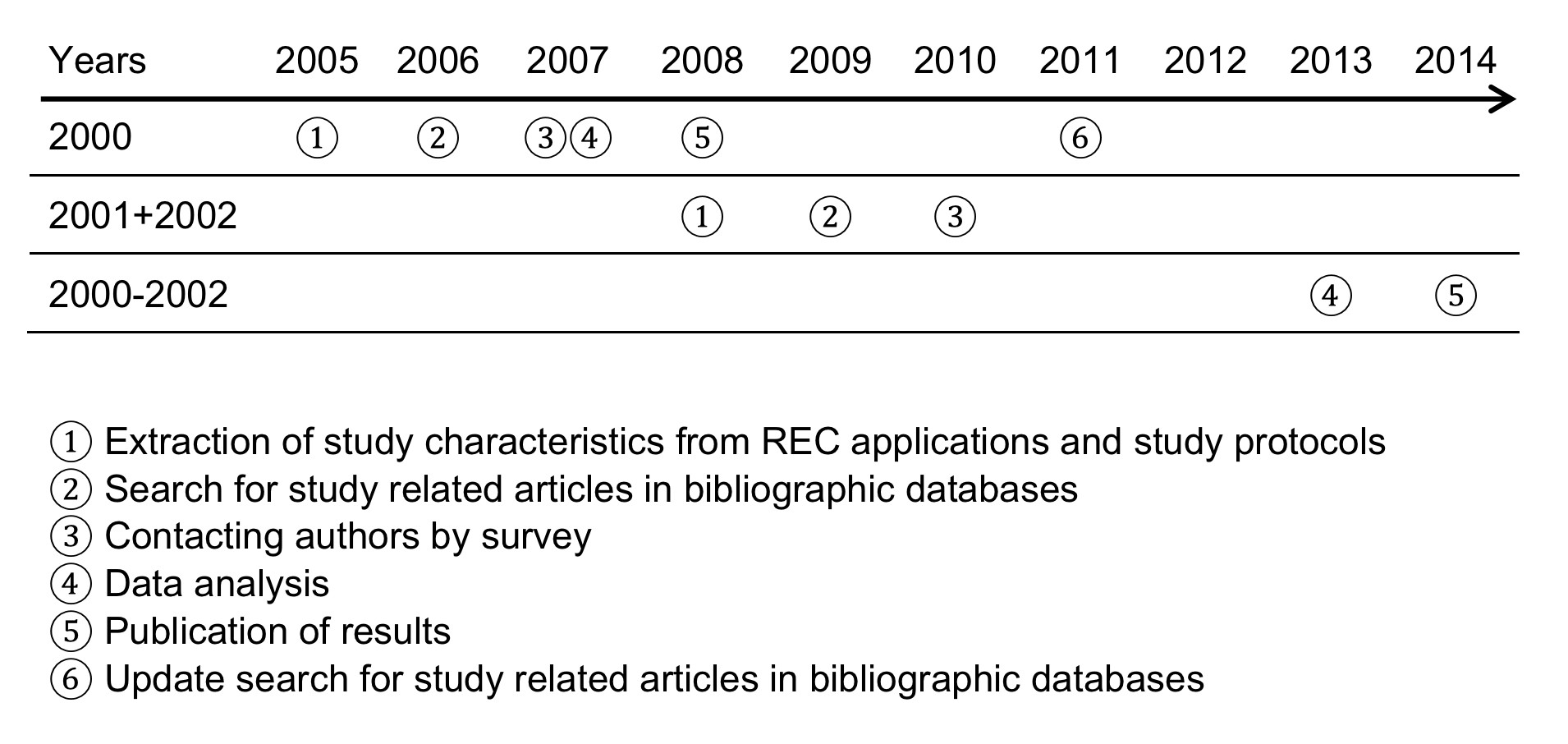

Supplement: S2 Fig — (TIFF) [file pone.0230797.s002.tiff]
