# Supplementary material for: A multi-state model analysis of the time from ethical approval to publication of clinical research studies
Source: PLoS One. 2020 Mar 27;15(3):e0230797. doi: 10.1371/journal.pone.0230797 (PMC7100954; doi:10.1371/journal.pone.0230797)
Supplement: S1 Appendix — (DOCX) [file pone.0230797.s003.docx]

**S1 Appendix: Extended statistical information**

# Observation pattern and censoring

- 1. **Retrospective event collection in a prospective cohort**

The present study is a prospective cohort study that sampled all studies with REC approval in baseline years 2000-2002. At fixed discrete follow-up time periods (i) the authors’ survey was sent out (2007 and 2010) and (ii) study-related articles were extensively searched (2006, 2009, and 2011); see Figure S2 for an overview. Both measures can be assumed to be *non-informative (or ignorable)* for the study progress and publication process, i.e., the examination periods do not depend on the event times of interest. At these examinations, the study progress (study completed or discontinued and the time to a potential first publication) was collected retrospectively for all studies initially included in the cohort. For the survey, the informative value on outcomes ‘study completed’ or ‘study discontinued’ is taken from the response received, while for the publication follow-up we assume that the (potentially right-censored) time to publication information could be retrieved for all studies initially sampled in the cohort. For those studies that were completed or stopped *after* the survey response and before the updated publication search, we only know their study status from the published article; otherwise their status is missing. Again, because of the fixed examination periods, we assume this loss to follow-up to also be non-informative. Being non-informative allows the observation process to be considered as *ignorable* in the likelihood-based analysis, i.e., the likelihood treating the observation process as non-random leads to the same inference as the correct likelihood (see e.g. Grüger et al. 1991).

- 1. **Censored event times and time units used**

In order to comply with the survey information received, we defined the study initiation date as the ***year*** of REC approval, the study completion date (if available) as the ***year*** in which data collection was completed, the study discontinuation date (if available) as the ***year*** in which the study was discontinued, and the study publication date as the ***year*** of electronic publication of the first manuscript presenting findings of the study.

Due to the finite length of observation not every transition from the initial state (here REC approval) to one or more of the states of interest (here study completion, study discontinuation, and publication) could be observed. Time to publication for studies without any resulting publication or where publication status was unclear were censored by the time the last bibliographic search was undertaken, which was either calendar year 2009 or 2011. This procedure is usually called **non-informative administrative right-censoring** (e.g., Andersen et al 1993, Andersen 2005), and it was done in both the two-state model analysis and the four-state model analysis.

The four-state model further accounts for the two intermediate events, study completion or study discontinuation, for which the following censoring mechanisms have been applied: Studies which were still ongoing at the time of the first survey response, i.e., 2007 (for REC approved studies from calendar year 2000), were included in the analysis as **interval-censored** between the time of survey response and the right-censored time to publication, i.e., in the likelihood inference (see section 2.2. below) they are given the “chance” to move into any of the intermediate states until publication or administrative censoring and are not assumed to be running until then. Studies with unclear study status (e.g. due to non-response to the 2007 or 2010 survey) or where the study status was known but its corresponding date was unavailable were included as **interval-censored** between study entry (date of REC approval) and the right-censored time to publication and are as such accounted for in the full likelihood of the four-state model (see section 2.2. below). We note here that as with administrative right-censoring the *independent censoring* *assumption* (Andersen et al 1993, Andersen 2005) also holds for interval-censoring as the interval is fixed and thus independent of the individual study processes. For background information on interval-censoring we refer readers to Andersen and Keiding (2002), Commenges (2002), and Van den Hout (2017), amongst others.

# Likelihood inference

- 1. **Two-state model**

Standard likelihood inference for right-censored data.

- 1. **Four-state model**

The estimation problem of interest is that of determining potential predictors of the timing of transitions within the research process from study initiation through completion/discontinuation to eventual publication. Typically, regression analysis for multi-state models is based on regression models for the transition intensities (hazards), and inference may be based on the Cox partial likelihood, allowing the baseline intensities to be left unspecified by ‘just’ accounting for the event cases conditional on being at risk for the respective event. However, with missing intermediate state information, this is no longer possible and one needs to consider a multi-state model approach based on the *full likelihood* (e.g., Joly et al 2002). In the appendix to his Master thesis, T Haag formulated corresponding likelihood contributions using transition probabilities and transition intensities from the four-state model for our observation pattern (as described above). Because of missing time to intermediate state information, the likelihood is only identifiable for certain functionals of the event history process. Therefore, we assume here the baseline transition intensities to be piecewise constant.

# A note on available software implementations

There are several software solutions available that can handle multi-state models in different situations. Alioum & Commenges (2001) published a FORTRAN-77 program to fit an arbitrary multi state model with piece-wise constant intensities. However, the program has a rather strong limitation, as it can only handle up to three time-intervals in which the transition intensities can be different. The R package msm (Jackson, 2011) is designed for fitting continuous-time Markov multi-state models to longitudinal data observed at arbitrary times by assuming constant or piece-wise constant intensities. While the package delivered good results in our tests with constant intensities, it did not converge reliably in tests with piecewise constant intensities and multiple intervals, leading to implausible results. The R package SmoothHazard allows to estimate irreversible illness-death models with possibly interval-censored data implementing a parametric approach with Weibull baseline intensities and a semi-parametric approach with M-splines approximation of baseline intensities in order to obtain smooth estimates of the transition intensities. It can, however, not be used for more general multi-state models.

As a suitable alternative to time-continuous multi-state models, discrete hazards models can be fitted to such time period data with the R package discSurv (Welchowski & Schmid) and by applying the glm function for generalized linear models (Tutz & Schmid 2016).

# References

Alioum, A and Commenges D. (2001). MKVPCI: a computer program for Markov models with piecewise constant intensities and covariates. Computer Methods and Programs in Biomedicine, 64:109–119. Program available from: <http://etudes.isped.u-bordeaux2.fr/BIOSTATISTIQUE/MKVPCI/US-Biostats-MKVPCI.htm>

Andersen, P.K., Borgan, Ø., Gill, R.D. & Keiding, N. (1993). Statistical Models Based on Counting Processes. New York: Springer.

Andersen P.K. (2005) Censored data. In: Encyclopedia of Biostatistics, Wiley.

Commenges D (2002). Inference for multi-state models from interval-censored data. Statistical Methods in Medical Research 11, 167–182.

Grüger J., Kay R., & Schumacher M. (1991). The validity of inferences based on incomplete observations in disease state models. Biometrics, 47(2), 595–605.

Jackson C. (2011). Multi-State Models for Panel Data: The msm Package for R. Journal of Statistical Software, 38(8), 1-28.

Jackson C. msm: Multi-state Markov and hidden Markov models in continuous time. <http://cran.r-project.org/web/packages/msm/index.html>. (first version released in 2002).

Joly P, Commenges D, Helmer C, and Letenneur L (2002). A penalized likelihood approach for an illness–death model with interval-censored data: application to age-specific incidence of dementia. Biostatistics 3, 433–443.

Touraine C, Gerds T, and Joly P. (2017). SmoothHazard: An R Package for Fitting Regression Models to Interval-Censored Observations of Illness-Death Models. Journal of Statistical Software, 79(7), 1-22.

Touraine C, Joly P, and Gerds TA. SmoothHazard: Estimation of Smooth Hazard Models for Interval-Censored Data with Applications to Survival and Illness-Death Models.

<https://cran.r-project.org/web/packages/SmoothHazard/index.html> (first version released in 2013)

Tutz G and Schmid M (2016) Modelling Discrete Time-to-event Data. New York: Springer.

Van den Hout A (2017) Multi-state survival models for interval-censored data. Chapman & Hall/CRC, Boca Raton, FL

Welchowski T and Schmid M. discSurv: Discrete Time Survival Analysis. <https://cran.r-project.org/web/packages/discSurv/index.html> (first version released in 2015).
