# Supplementary material for: A multi-state model analysis of the time from ethical approval to publication of clinical research studies
Source: PLoS One. 2020 Mar 27;15(3):e0230797. doi: 10.1371/journal.pone.0230797 (PMC7100954; doi:10.1371/journal.pone.0230797)
Supplement: S1 Table — (DOCX) [file pone.0230797.s004.docx]

S1 Table. Estimated covariate effects with their 95% confidence intervals from a logistic regression model (see Sensitivity analysis in main manuscript).

|  | **Odds ratio** | **95% CI** |
| --- | --- | --- |
| **Approved 🡪 Published** |  |  |
| Log sample size | 1.069 | [0.944, 1.210] |
| RCT vs. other | 0.990 | [0.684, 1.432] |
| Funding: Commercial vs. non-commercial | 0.157 | [0.088, 0.279] |
| Funding: Unstated vs. non-commercial | 0.169 | [0.110, 0.259] |
| Industry: Involved vs. not involved | 2.795 | [1.566, 4.991] |
| Primary outcome: Yes vs. no | 1.723 | [1.154, 2.573] |
| Collaboration: National multi-centre vs. national single-centre | 0.824 | [0.489, 1.390] |
| Collaboration: International multi-centre vs. national single-centre | 0.520 | [0.327, 0.826] |
