# Supplementary material for: A multi-state model analysis of the time from ethical approval to publication of clinical research studies
Source: PLoS One. 2020 Mar 27;15(3):e0230797. doi: 10.1371/journal.pone.0230797 (PMC7100954; doi:10.1371/journal.pone.0230797)
Supplement: S2 Table — (DOCX) [file pone.0230797.s005.docx]

S2 Table. Estimated covariate effects with their 95% confidence intervals from separate logistic regression models (see Sensitivity analysis in main manuscript).

|  | **Odds ratio** | **95% CI** |
| --- | --- | --- |
| **Approved 🡪 Completed** |  |  |
| Log sample size | 1.025 | [0.897, 1.172] |
| RCT vs. other | 0.812 | [0.544, 1.211] |
| Funding: Commercial vs. non-commercial | 0.267 | [0.147, 0.485] |
| Funding: Unstated vs. non-commercial | 0.405 | [0.262, 0.625] |
| Industry: Involved vs. not involved | 3.554 | [1.933, 6.535] |
| Primary outcome: Yes vs. no | 1.599 | [1.052, 2.431] |
| Collaboration: National multi-centre vs. national single-centre | 0.844 | [0.478, 1.492] |
| Collaboration: International multi-centre vs. national single-centre | 0.650 | [0.407, 1.038] |
|  |  |  |
| **Approved 🡪 Discontinued** |  |  |
| Log sample size | 0.876 | [0.739, 1.039] |
| RCT vs. other | 2.384 | [1.473, 3.858] |
| Funding: Commercial vs. non-commercial | 2.713 | [1.377, 5.343] |
| Funding: Unstated vs. non-commercial | 1.813 | [1.020, 3.223] |
| Industry: Involved vs. not involved | 0.591 | [0.302, 1.156] |
| Primary outcome: Yes vs. no | 0.992 | [0.583, 1.690] |
| Collaboration: National multi-centre vs. national single-centre | 0.846 | [0.424, 1.688] |
| Collaboration: International multi-centre vs. national single-centre | 1.112 | [0.622, 1.989] |
|  |  |  |
| **Completed 🡪 Published** |  |  |
| Log sample size | 1.067 | [0.944, 1.207] |
| RCT vs. other | 0.905 | [0.625, 1.310] |
| Funding: Commercial vs. non-commercial | 0.147 | [0.081, 0.266] |
| Funding: Unstated vs. non-commercial | 0.213 | [0.140, 0.324] |
| Industry: Involved vs. not involved | 3.543 | [1.942, 6.462] |
| Primary outcome: Yes vs. no | 1.508 | [1.011, 2.250] |
| Collaboration: National multi-centre vs. national single-centre | 0.750 | [0.447, 1.259] |
| Collaboration: International multi-centre vs. national single-centre | 0.436 | [0.273, 0.696] |
|  |  |  |
| **Discontinued 🡪 Published** |  |  |
| Log sample size | 1.010 | [0.738, 1.383] |
| RCT vs. other | 1.420 | [0.574, 3.516] |
| Funding: Commercial vs. non-commercial | 1.163 | [0.427, 3.168] |
| Funding: Unstated vs. non-commercial | -- | -- |
| Industry: Involved vs. not involved | 0.385 | [0.129, 1.151] |
| Primary outcome: Yes vs. no | 3.383 | [0.849, 13.489] |
| Collaboration: National multi-centre vs. national single-centre | 2.384 | [0.571, 9.958] |
| Collaboration: International multi-centre vs. national single-centre | 2.913 | [0.819. 10.364] |
