# Supplementary material for: A multi-state model analysis of the time from ethical approval to publication of clinical research studies
Source: PLoS One. 2020 Mar 27;15(3):e0230797. doi: 10.1371/journal.pone.0230797 (PMC7100954; doi:10.1371/journal.pone.0230797)
Supplement: S3 Table — (DOCX) [file pone.0230797.s006.docx]

**S3 Table.** Four-state model observation cases with coding of potential censoring times and corresponding counts (#) and percentages (%) in the data set. “Interval left”: left boundary of censoring interval (with individual left censoring time E_i_); “Interval right”: right boundary of censoring interval (with individual right-censoring time R_i_). T_i_ individual time to intermediate event, T_Pi_ individual time to publication, C right-censored publication time

| **Case** | **Description** | **Transition in four-state-model** | **Indicator** | | | **Event time** | | | **Condition** | **N** | **%** |
| --- | --- | --- | --- | --- | --- | --- | --- | --- | --- | --- | --- |
|  |  |  | **Comp-leted** | **Discon-tinued** | **Pub-lished** | **Interval left** | **Interval right** | **Time-to-publication** |  |  |  |
| 1 | Study ongoing by time of survey, Interval-censored from year of survey to right-censored publication year | 0 🡪 0 | 0 | 0 | 0 | R_i_ | C_i_ | C_i_ | R_i_ <= C_i_ | 41 | 5.1% |
| 2 | Study status unknown, not published, Interval-censored from year of entry to right-censored publication year | 0 🡪 0 | 0 | 0 | 0 | E_i_ | C_i_ | C_i_ | E_i_ < C_i_ | 61 | 7.6% |
| 3 | Known completion year, right-censored publication year | 0 🡪 1 | 1 | 0 | 0 | T_i_ | T_i_ | C_i_ | T_i_ <= C_i_ | 48 | 6.0% |
| 4 | Interval-censored completion year (status known from survey), right-censored publication year | 0 🡪 1 | 1 | 0 | 0 | E_i_ | R_i_ | Ci | E_i_ < R_i_ <= C_i_ | 165 | 20.5% |
| 5 | Known discontinuation year, right-censored publication year | 0 🡪 2 | 0 | 1 | 0 | T_i_ | T_i_ | C_i_ | T_i_ <= C_i_ | 11 | 1.4% |
| 6 | Interval-censored discontinuation year (status known from survey), right-censored publication year | 0 🡪 2 | 0 | 1 | 0 | E_i_ | R_i_ | Ci | E_i_ < R_i_ <= C_i_ | 85 | 10.5% |
| 7 | Known completion year, publication year observed | 1 🡪 3 | 1 | 0 | 1 | T_i_ | T_i_ | T_Pi_ | T_i_ <= T_Pi_ | 74 | 9.2% |
| 8 | Interval-censored completion year (status known from survey), publication year observed | 1 🡪 3 | 1 | 0 | 1 | E_i_ | R_i_ | T_Pi_ | E_i_ < R_i_ <= T_Pi_ | 289 | 35.9% |
| 9 | Known discontinuation year, publication year observed | 2 🡪 3 | 0 | 1 | 1 | T_i_ | T_i_ | T_Pi_ | T_i_ <= T_Pi_ | 4 | 0.5% |
| 10 | Interval-censored discontinuation year (status known from survey), publication year observed | 2 🡪 3 | 0 | 1 | 1 | E_i_ | R_i_ | T_Pi_ | E_i_ < R_i_ <= T_Pi_ | 28 | 3.5% |
